# hNIS-based imaging to monitor treatment with the novel oncolytic virus CF33-hNIS-antiPDL1 in humans with advanced triple negative breast cancer

**DOI:** 10.3389/fonc.2025.1565244

**Published:** 2025-08-25

**Authors:** Jamie Rand, Dave Yamauchi, Shyambabu Chaurasiya, Jianying Zhang, Supriya Deshpande, Leslie Chong, Amanda Seiz, Hans Meisen, Yuman Fong, Yuan Yuan

**Affiliations:** ^1^ Department of Surgery, City of Hope Comprehensive Cancer Center, Duarte, CA, United States; ^2^ Department of Radiology, City of Hope Comprehensive Cancer Center, Duarte, CA, United States; ^3^ Department of Statistics, City of Hope Comprehensive Cancer Center, Duarte, CA, United States; ^4^ Imugene Limited, Sydney, NSW, Australia; ^5^ Department of Translational Development, City of Hope Comprehensive Cancer Center, Duarte, CA, United States; ^6^ Department of Medicine, Cedars Sinai Medicine, Los Angeles, CA, United States

**Keywords:** triple-negative breast cancer, oncolytic virus, PET, SPECT imaging, Tc-99m, noninvasive imaging

## Abstract

**Background:**

Triple-negative breast cancer (TNBC) is clinically aggressive. CF33-hNIS-antiPDL1, an oncolytic orthopoxvirus, shows robust anti-cancer activity in TNBC xenografts in mice. CF33-hNIS-antiPDL1-infected tumor cells express functional human sodium iodide symporter (hNIS) and are visible by single-photon emission computed tomography (SPECT) or positron emission tomography (PET). We evaluated the ability of virus-encoded hNIS to track OV in mice using PET imaging and in a phase I study in TNBC patients using SPECT. The aim of this first-in-human study was to determine imageability and safety of intratumoral (IT) CF33-hNIS-antiPDL1 injections.

**Methods:**

Imageability of CF33-hNIS-antiPDL1 was first assessed in mice bearing human xenografts. Virus or PBS-treated mice were imaged using a PET scanner. For the first-in-human trial, 9 patients were enrolled in this phase I, single-center, single-arm trial from October 2021 to August 2023. Key eligibility criteria included unresectable/metastatic TNBC; progressed on at least 2 prior chemotherapies; ECOG 0-2; RECIST 1.1 measurable disease; and at least one tumor amenable to repeated IT injections. Eligible patients received CF33-hNIS-antiPDL1 IT at 1 of 6 assigned dose levels (ranging from 1 × 105 PFU to 3 x 108 PFU) on days 1 and 15 of each 28-day cycle for 3 treatment cycles. SPECT whole-body imaging was performed using technetium-99 at cycle 1 day 8.

**Results:**

All mice treated with the virus showed clear PET signal from tumors whereas no signal was observed in PBS-treated mice. In the phase I study, 7 of 9 patients (78%) showed uptake at the injection site on SPECT imaging at C1D8. Five of 5 patients (100%) with injection sites at metastatic subcutaneous nodules, intramuscular masses, or axillary lymph nodes, and 2/4 patients (50%) with injection sites at matted dermal metastatic lesions had uptake at injected lesions.

**Conclusion:**

SPECT imaging successfully showed enhancement at the injected lesions in 78% of patients treated with CF33-hNIS-antiPDL1, even at low doses of the oncolytic virus (OV), suggesting local viral replication and hNIS expression. This is the first report of hNIS-based imaging to track oncolytic poxvirus replication in humans. This technology holds promise for noninvasive tracking of systemically administered OVs and other therapies.

**Clinical trial registration:**

https://www.clinicaltrials.gov/study/NCT05081492, identifier NCT05081492.

## 1 Introduction

Breast cancer is the most commonly diagnosed cancer worldwide (1). According to the American Cancer Society, around 310,720 new cases of invasive breast cancer will be diagnosed among women, and 56,500 women will die from the disease in the US in 2024 (2). Triple-negative breast cancer (TNBC) is clinically aggressive, has the highest death rate, and worst overall survival (2). There is an imperative need for early detection and novel treatment options to reduce deaths due to breast cancer.

Oncolytic viruses (OVs) that specifically target tumor cells are evolving as promising immunotherapeutic agents for the treatment of cancers. Engineered OVs have been widely tested in preclinical studies and clinical trials (3). Talimogene latherparepvec (T-Vec) is the only FDA-approved OV immunotherapy for metastatic melanoma that uses an engineered herpes simplex virus encoding human GM-CSF (4).

Proving that OVs target and amplify specifically at tumor sites is a goal of human trials. Thus, tracking viral activity is essential to correlate to tumor responses and toxicities. Invasive methods, such as blood and tissue sample collection, biopsy, and histopathological analysis, have been used for monitoring such responses. Noninvasive strategies are clinically beneficial as these provide spatiotemporal information to identify tumor sites targeted by viruses and comprehensively understand the effects of viroimmunotherapy using molecular imaging. Human sodium iodide symporter (hNIS) is a transmembrane protein expressed on thyroid follicular cells that transports iodide for thyroid hormone synthesis. Ectopic hNIS expression has been used to facilitate the transport of radiotracers such as radioiodine ^123^I or technetium-pertechnetate (Tc-99m) (5, 6). The hNIS gene can be tracked by positron emission tomography (PET) and single-photon emission computed tomography (SPECT) imaging studies. Various *in vitro* and *in vivo* studies have shown the utility of noninvasive NIS imaging to verify targeted vector infection in breast, prostate, ovarian, and thyroid cancers (7).

We previously developed CF33-hNIS and CF33-hNIS-antiPDL1, chimeric pox viruses encoding hNIS, to allow for noninvasive imaging, comprehensive tracking of real-time virus distribution and replication, as well as synergistic tumor cell death due to radioisotope uptake (8). We have successfully imaged CF33-hNIS by PET in mouse cancer models (8–10). In addition to hNIS, CF33-hNIS-antiPDL1 contains an expression cassette for soluble single chain variable fragment (scFV) against PD-L1, i.e., PD-L1 scFv. We recently performed preclinical pharmacology studies and showed the robust antitumor efficacy and safety of CF33-hNIS-antiPDL1 against TNBC using xenograft and syngeneic mouse models (11).

Here, we present the results from a first-in-human trial of CF33-hNIS-antiPDL1 in metastatic TNBC (mTNBC). For the first time, we have successfully tracked an oncolytic vaccinia virus in humans using SPECT imaging.

## 2 Methods

### 2.1 Pre-clinical studies

#### 2.1.1 Cell lines

African green monkey kidney cells CV-1 and human liver cancer cells Hep3B were purchased from ATCC (Manassas, USA). CV-1 and HEP3B cells were cultured in DMEM and RPMI1640 medium, respectively. Media were supplemented with 10% FBS, 2 mmol/L L-glutamine, and 100 U/mL penicillin–streptomycin. All cell culture supplies were purchased from Corning (Corning, New York). Cells were maintained in a humidified incubator at 37 °C and 5% CO_2_.

#### 2.1.2 Virus

CF33 is a chimeric poxvirus constructed from recombination among nine strains/species of poxviruses as described previously (12). CF33 was engineered to create CF33-hNIS-antiPD-L1 as described by Chaurasiya et al. (10). Briefly, homologous recombination technique was used to insert expression cassettes for hNIS and PD-L1 scFv in the CF33 genome. The viral genes J2R and F14.5L were replaced by hNIS and PD-L1 scFv expression cassettes, respectively. The recombinant virus was selected using plaque purification in the presence of drug cocktail (xanthine, hypoxanthine, and mycophenolic acid) and was verified by PCR and sequencing. The virus was amplified in CV-1 cells and purified using sucrose gradients and used in preclinical studies. For clinical studies, clinical lots of the virus were manufactured under current good manufacturing practice (CGMP) conditions as described previously (11).

#### 2.1.3 Tumor model

All animal studies were conducted under a City of Hope Institutional Animal Care and Use Committee approved protocol (#15003) in compliance with National Institute of Health’s guideline for care and use of laboratory animals. Mouse studies were performed to determine if quantification of PET scanning correlates with viral burden. *In vitro* studies showed comparable anti-tumor activity in both liver and breast cancer models. The described imaging-related mouse studies were performed using the liver cancer model. The human liver cancer cell line (HEP-3B) was used to generate subcutaneous flank tumors in athymic nude female mice aged 6–8 weeks. Briefly, HEP-3B cells were cultured in T225 flasks in RPMI 1640 supplemented with 10% Fetal Bovine Serum (FBS). Cells were harvested using trypsin at approximately 80% confluency and washed twice with 1X Phosphate Buffer Saline (PBS). Cells were counted and resuspended in 1-part Matrigel to 1-part PBS at a concentration of 5x10^6^ cells/50 µL volume. Cells were retained on ice until the time of injection. Mice were anesthetized using isoflurane, and sterile eye ointment was applied to their eyes to avoid dryness. Then 50 µL of cells were slowly injected subcutaneously per mouse to generate a single flank tumor per mouse. Ear-tags were applied for identification. Nineteen days after cell injection, all mice had tumors in the range of 100 mm^3–^300 mm^3^. Mice were injected intratumorally with 50 µL of CF33-hNIS-antiPDL1 at one of three dose levels (1x10^4^, 1x10^5^, or 1x10^6^ plaque forming units (PFU)) or control (1X PBS). After virus injection, mice (n=6 for each virus dose, and n=3 for PBS) were monitored daily.

#### 2.1.4 Positron Emission Tomography imaging

Three mice from each virus-treated group and one mouse from the control group were prepared for PET imaging. One week before imaging, mice were fed with water containing 5 mg/L levothyroxine to minimize uptake of ^124^I by the thyroid gland and monitored daily. Forty-eight hours post virus injection, mice were injected with 150 µCi of ^124^I in 100 µL of PBS and imaged using PET imaging. Each mouse was imaged for 2 hours after the ^124^I injection.

#### 2.1.5 Determination of virus titer in tumor

Three mice from each virus-treated group and 2 from the control group were humanely euthanized using CO_2_ at the time of PET imaging. Tumors and other organs were harvested and immediately frozen in liquid nitrogen. Organs and tumors were stored at -80 °C until use. Tumors were weighed, and lysates were prepared in 0.5 mL PBS supplemented with protease inhibitors. Finally, a standard plaque assay was performed on the tumor lysates to determine the virus titer. A lab stock of CF33-hNIS-antiPDL1 was used as a positive control in the plaque assay.

### 2.2 Clinical trial subjects and study design

**Figure 1:**
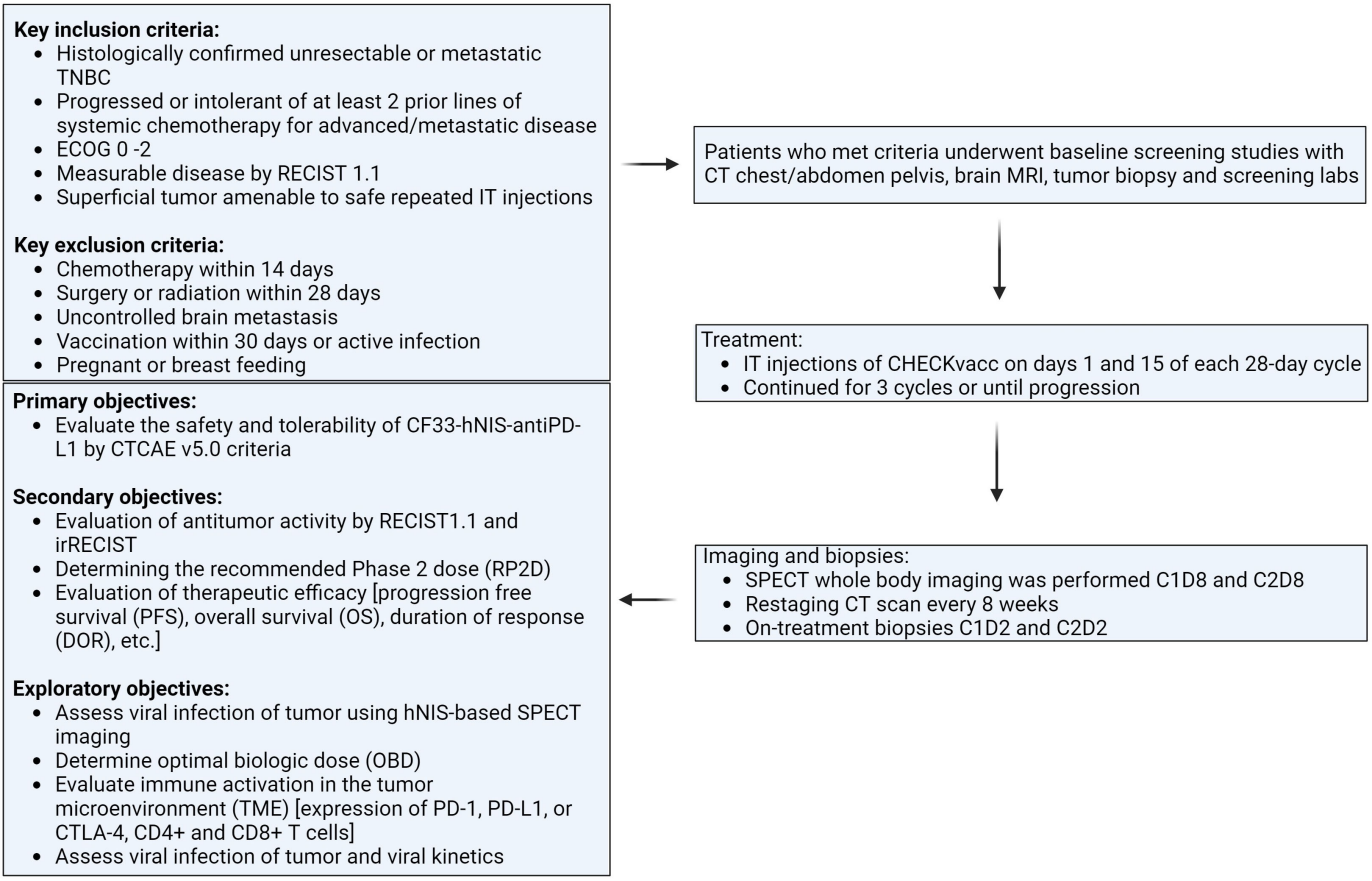
Flowchart showing the study design of the clinical trial.

This is a first-in-human phase I, single-center, single-arm clinical trial evaluating the safety and tolerability of CF33-hNIS-antiPDL1 intratumoral (IT) injection in patients with metastatic TNBC (NCT05081492). The study was approved by the City of Hope Institutional Review Board (IRB), and all subjects provided written consent (IRB# 21094). Study schema is shown in **Figure 1**.

Key eligibility criteria were: 1) Histologically confirmed unresectable or mTNBC, defined as estrogen receptor (ER) and progesterone receptor (PR) ≤ 10% by IHC, and human epidermal growth factor receptor 2 (HER2) negative per ASCO/CAP guidelines; 2) Patients must have progressed or been intolerant of at least 2 prior lines of systemic chemotherapy for advanced/metastatic disease; 3) ECOG 0 – 2; 4) Measurable disease by RECIST 1.1; 5) Must have a superficial tumor (cutaneous, subcutaneous), breast lesion or nodal metastases amenable to safe repeated IT injections according to the treating physician and interventional radiologist review.

Key exclusion criteria were: 1) Chemotherapy within 14 days; 2) Surgery or radiation within 28 days; 3) Uncontrolled brain metastasis; 4) Vaccination within 30 days; 5) Active infections; 6) Pregnant or breastfeeding.

Following a Phase I Queue (IQ) 3 + 3 design, eligible patients received IT CF33-hNIS-antiPDL1 at 1 of 6 assigned dose levels (1 × 10^5^, 3 x 10^5^, 1 x 10^6^, 1 x 10^7^, 1 x 10^8^, or 3 x 10^8^ PFU) on days 1 and 15 of each 28-day cycle for a total of 3 cycles of treatment. The first 3 subjects were enrolled sequentially, each receiving 1 injection and completing a 4-week safety period before the next was treated. At least 3 subjects completed the first evaluation period (or have DLT) before a dose escalation decision was made. DLT was defined as any Grade 3 or 4 toxicities possibly related to CF33-hNIS-antiPDL1 (excluding some Grade 3 injection site reactions, rashes, fatigue, GI symptoms, and transient lab abnormalities).

The primary objectives of this study were to evaluate the safety and tolerability of CF33-hNIS-antiPDL1 by CTCAE v5.0 criteria. Secondary objectives included evaluation of antitumor activity by RECIST1.1 and irRECIST, determining the recommended Phase II dose (RP2D, and evaluating therapeutic efficacy [progression-free survival (PFS) and overall survival (OS)]. Exploratory objectives included assessing viral infection of the tumor using hNIS-based SPECT imaging, viral shed, antiviral immune activation [expression of PD-1, PD-L1, or CTLA-4 and CD8+ T-cells], and determining optimal biologic dose (OBD).

Noninvasive SPECT whole-body imaging was performed 7 days after administration of CF33-hNIS-antiPDL1 at cycle 1, day 8 (C1D8), and at cycle 3, day 2 (C3D2).

#### 2.2.1 SPECT imaging studies

Ten millicurie of Tc-99m pertechnetate was intravenously injected. Approximately fifteen minutes after injection, anterior and whole-body camera views were obtained. Shortly thereafter, axial SPECT and CT images of the area of interest were obtained, which were then fused and reconstructed in the coronal and sagittal planes.

### 2.3 Statistics

*In vivo*, one-way ANOVA was used to compare virus titers in the tumors of mice treated with different doses of virus. A P value of 0.05 or less was considered statistically significant. GraphPad Prism Software (GraphPad Software, La Jolla, CA, USA) was used for statistical analyses and data visualizations. Patient demographic and baseline characteristics, including age, gender, medical history, and prior therapy, were summarized using descriptive statistics (mean, standard deviation, and median (range) for continuous variables; count and percentage for categorical variables).

## 3 Results

### 3.1 Pre-clinical hNIS imaging studies

**Figure 2:**
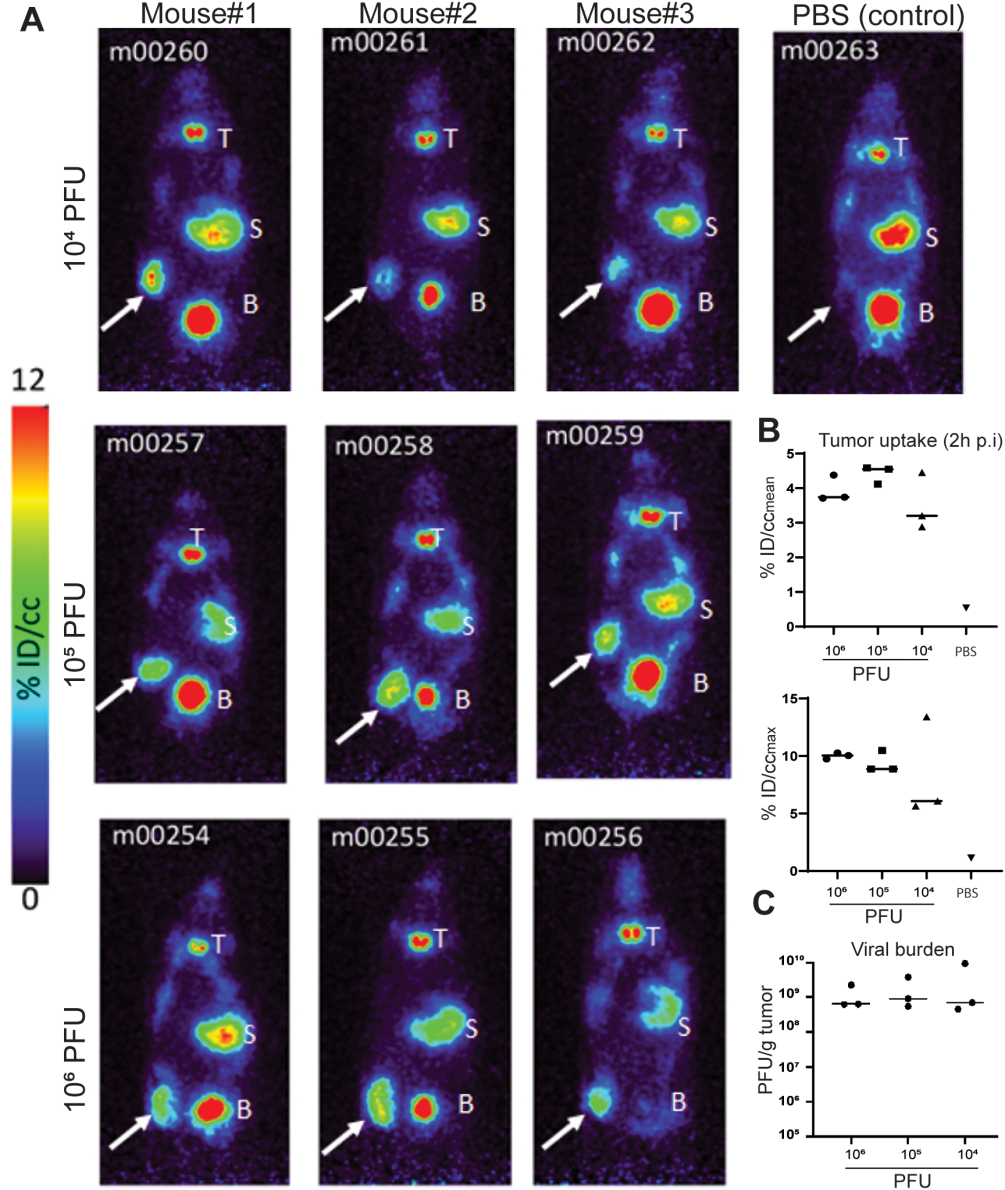
PET imaging results. **(A)** PET images of virus and PBS-treated mice. Mice were injected IT with virus or PBS. Forty-eight hours post-virus injection, ^124^I was injected through tail-vein, and 2 hours later, mice were imaged using a PET scanner. White arrows point to tumors. T, thyroid; S, stomach; B, urinary bladder. Note that the high signals in the thyroid, stomach, and bladder are due to endogenous NIS expression in these organs. **(B)** Quantification of ^124^I uptake in tumors 2 hours post-injection (p.i.). The radioactivity concentration (percent of the injected dose/cubic centimeter, %ID/cc) as both the mean concentration (%ID/cc_mean_) across the region of interest and the maximum concentration area within the tumor (%ID/cc_MAX_) are presented. **(C)** Viral burden in tumors at the time of imaging.

Prior to initiating the first-in-human trial, CF33-hNIS-antiPDL1 was extensively studied in mouse models for safety and anti-tumor efficacy (10, 13–17). Forty-eight hours post-virus treatment, mice were injected with ^124^I and PET imaging was performed. PET imaging showed significant uptake at the tumor in all mice injected with CF33-hNIS-antiPDL1 at all dose levels (**Figures 2A, B**), compared to minimal uptake in the PBS-treated control group. The viral burden in the tumor did not differ between dose levels (**Figure 2C**), suggesting that the virus replicated rapidly within the tumors.

### 3.2 Patient accrual and demographics

From October 2021 to August 2023, nine patients were enrolled in this phase I first-in-human clinical trial. Baseline demographics are shown in **Table 1**. The median age of participants was 64 (range: 48-81). The majority of patients were White (56%). All patients (100%) had stage IV disease. All patients were heavily pre-treated, with a median number of prior systemic therapy lines for mTNBC of 4 (range: 2-8). In addition to these treatments, most patients also previously received locoregional therapy, including radiation and/or surgery, and systemic therapy prior to progression to stage IV disease. The sites injected with the OV were axillary lymph node metastasis in one patient, intramuscular mass in one patient, matted dermal metastasis in four patients, and dermal/subcutaneous nodules in three patients. This study is registered at ClinicalTrials.gov under the number NCT05081492.

**Table 1:** Demographics of study population.

Characteristic	Number (%)
Gender	
Female	9 (100%)
Age: Median (Range)	64 (48-81)
Race	
White	5 (56%)
Black	1 (11%)
East Asian	2 (22%)
South Asian	1 (11%)
Number of prior lines of therapy for mTNBC: Median (Range)	4 (2-8)
Site injected with oncolytic virus	
Lymph node:	1 (11%)
Intramuscular mass:	1 (11%)
Dermal/subcutaneous nodules:	3 (33%)
Matted dermal disease:	4 (44%)
Cancer staging	
Stage IV	9 (100%)
Prior immune checkpoint blockade:	6 (67%)

### 3.3 CF33-hNIS-antiPDL1 treatment and safety data

Nine patients received at least one dose of IT CF33-hNIS-antiPDL1 for mTNBC at one of the first three doses (1x10^5^, 3x10^5^, 1x10^6^ PFU). All patients underwent at least one whole-body SPECT imaging study using Tc-99m. SPECT imaging studies were performed at cycle 1, day 8 (C1D8), and cycle 3, day 2 (C3D2). One patient underwent SPECT imaging studies at C1D8 and C3D2, while the other eight patients were off-trial prior to the second imaging study due to disease progression or patient preference. The treatment was well tolerated, with no dose-limiting toxicities.

### 3.4 SPECT imaging and patient outcomes

**Figure 3:**
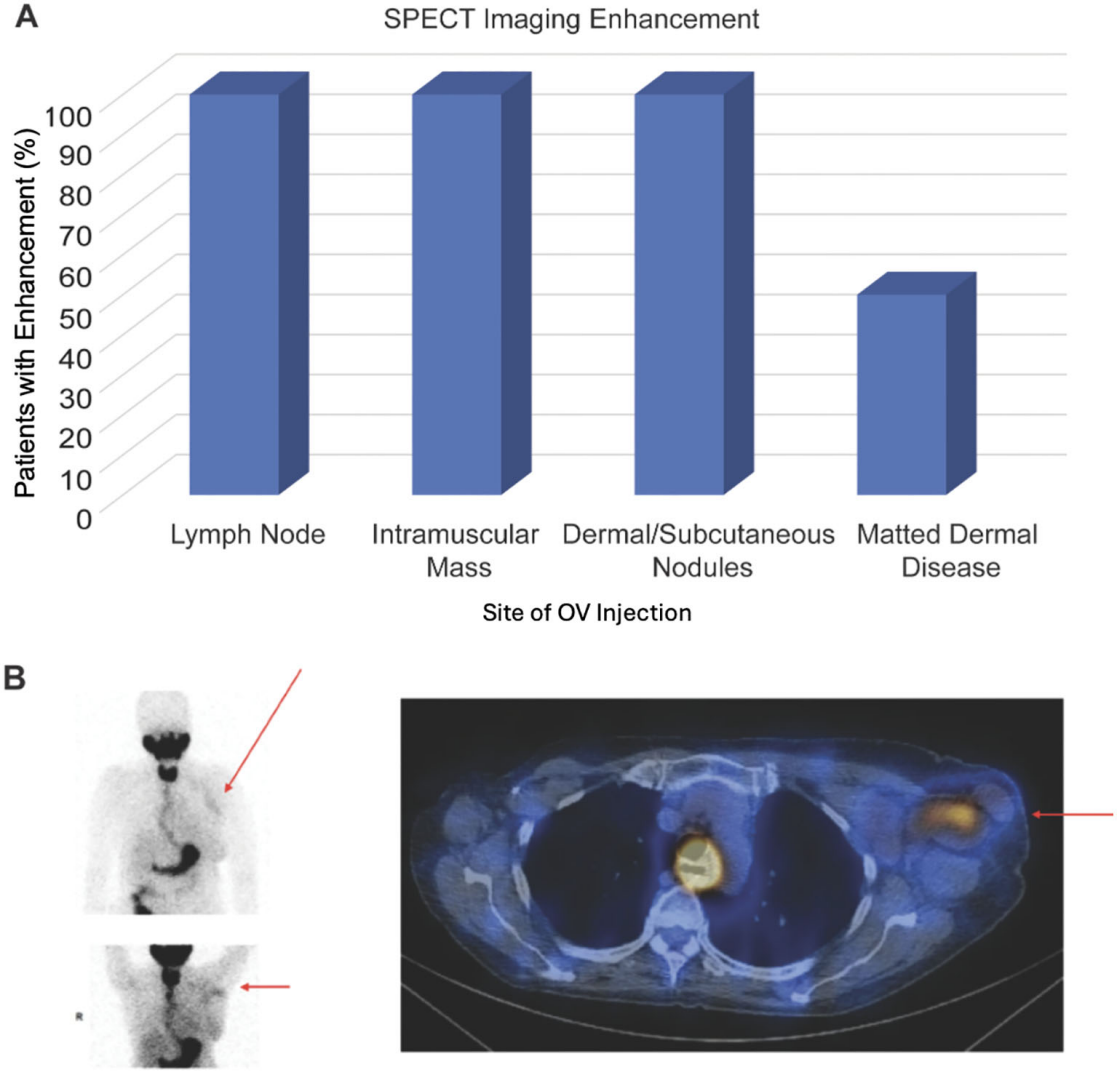
SPECT imaging results. **(A)** Graph showing percentage of patients that had enhancement on SPECT imaging following treatment with CF33-hNIS-antiPDL1 for each type of tumor injection site. **(B)** Patient 1 received CF33-hNIS-antiPDL1 at dose level 2 (3x10^5^ PFU). Injected lesion was left axillary lymph node. SPECT imaging at C1D8 showed enhancement of injected lymph node (red arrows).

Of the 9 patients treated with IT CF33-hNIS-antiPDL1, 78% (7 of 9) showed uptake of Tc-99m at the injected lesion on SPECT imaging, suggesting viral replication in the tumor at the site of injection. In the two patients without any uptake, the injected lesions were matted dermal metastasis. Fifty percent (2 of 4) patients with matted dermal disease had enhancement on SPECT imaging. All patients (5 of 5) with injection sites at lymph nodes, intramuscular mass, or dermal/subcutaneous nodules had enhancement on SPECT imaging (**Figure 3A**).

The two patients with the most robust uptake on SPECT imaging studies are presented here in more detail.

Patient 1

Representative images from C1D8 SPECT imaging study for the first patient are shown in **Figure 3B**. This patient initially presented in 2006 with stage IIB right breast cancer (ER+, HER2-) and underwent a right partial mastectomy and axillary lymph node dissection, adjuvant chemotherapy, adjuvant radiation, and endocrine therapy for five years. She did well until 2021 when she presented with left axillary pain and had a workup that showed bilateral breast masses and abnormal left axillary lymph nodes. Biopsies of these areas showed right TNBC, left ER+, HER2- breast cancer, and left axillary lymph node metastasis consistent with TNBC. Staging scans showed left axillary, supraclavicular, and mediastinal lymphadenopathy suspicious for metastasis. Endobronchial ultrasound and mediastinal lymph node biopsy confirmed mTNBC. In August 2021, she was started on xeloda and pembrolizumab. PET/CT in December 2021 showed disease progression; thus, systemic therapy was switched to sacituzumab govitecan. Disease progressed on staging scans in March 2022, and she was enrolled in this clinical trial. She received her first injection of CF33-hNIS-antiPDL1 into the left axillary lymph node using ultrasound guidance on April 7, 2022, at dose level two (3x10^5^). The patient received four injections of OV. Staging scans on May 26, 2022, showed disease progression both locally and at distant sites, thus she came off trial. Her SPECT imaging scans on C1D8 showed uptake at the left axillary lymph nodes (**Figure 3B**), suggesting viral replication at the injected tumor site in the left axillary lymph nodes.

Patient 2

**Figure 4:**
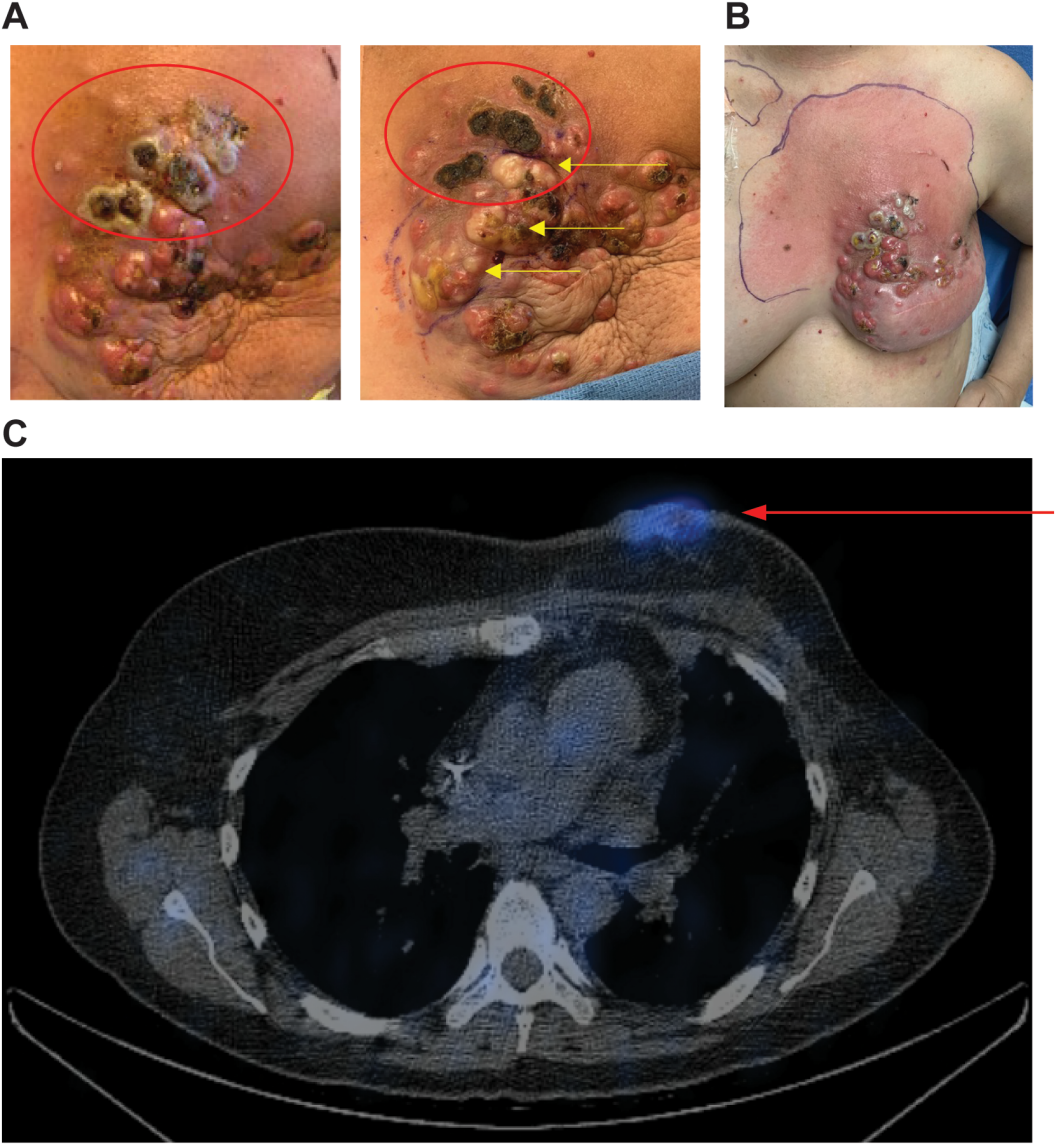
Robust injection site reaction and uptake on SPECT imaging following treatment with dose level 3. **(A)** Patient 2 received CF33-hNIS-antiPDL1 at dose level 3 (1x10^6^ PFU). Injected lesions were superior left chest wall nodules. Injected nodules demonstrated necrosis on clinical exam (red ovals). The image on the left shows C1D11, right shows C1D21 with continued necrosis of superior injected lesions and blanching of lesions inferior to that following injection #2 into the inferior lesions (yellow arrows). **(B)** Robust injection site reaction shown on C1D8 with surrounding erythema. **(C)** SPECT imaging showed enhancement of injected nodules (red arrow).

SPECT images for the second patient are shown in **Figure 4C**. This patient initially presented in 2014 at age 39 with *de novo* stage IV ER+, HER2- breast cancer with metastasis to the left axillary lymph nodes and left rib. She was initially started on ovarian suppression, denosumab, and endocrine therapy, which controlled her disease well until June 2020, when she had disease progression at the breast. She then started fulvestrant and cyclin-dependent kinase (CDK) 4/6 inhibitors but did not tolerate multiple CDK 4/6 inhibitors and subsequently had disease progression. She then started albumin-bound paclitaxel, followed by eribulin, with continued disease progression. PET/CT scans in December 2020 showed no evidence of distant metastasis, but there was continued progression at the breast, and she underwent mastectomy with sentinel lymph node biopsy with pathology showing grade 3 invasive lobular carcinoma 13.5 cm in size with dermal invasion, margins positive, 3/3 positive lymph nodes, and biomarkers now triple negative. She subsequently underwent radiation to the left chest wall and nodal basins. Four months after radiation, she developed new left chest wall dermal nodules, which were biopsied and consistent with TNBC. Staging scans showed new hepatic metastases and bony metastases. She was started on carboplatin and gemcitabine, complicated by profound cytopenias. She was switched to sacituzumab govitecan with disease progression. She was then switched to adriamycin and cyclophosphamide with progression, followed by fam-trastuzumab deruxtecan-nxki with continued progression. She was subsequently enrolled in the CF33-hNIS-antiPDL1 clinical trial at dose level three (1x10^6^). She had a robust clinical response to her first injection, with pain and erythema throughout the left chest wall. On clinical exam, she had necrosis of the injected lesions (**Figures 4A, B**). She was admitted to the hospital, started on empiric antibiotics, and underwent extensive testing, which ruled out infectious causes. The leading cause for her erythema and pain in the left chest wall was a robust injection site reaction. The reaction subsequently resolved without further treatment. Her second dose of OV was delayed by one week to allow for recovery and was dose reduced per protocol (3x10^5^). She tolerated the second dose well, but her disease progressed at the non-injected sites of the chest wall and sites of distant metastasis, and she came off-trial. SPECT imaging scans at C1D8 showed enhancement at the injected left chest wall dermal nodules (**Figure 4C**).

## 4 Discussion

As immunotherapy treatments become increasingly widespread for cancer treatment, the ability to monitor these treatments using noninvasive techniques has become more important. hNIS-based imaging in mouse models has been successful in monitoring viral replication and tracking *in vivo* using noninvasive imaging techniques. We and others have previously demonstrated the usefulness of hNIS for tracking OVs in preclinical studies (8, 10, 18). However, there have been limited studies showing the success of hNIS-based imaging studies to track oncolytic virotherapy in humans.

Most OV clinical trials involve melanoma and GI cancers (19). Previous imaging studies in patients treated with OVs expressing NIS have successfully confirmed infection of macroscopic tumor deposits (20, 21). However, only a handful of publications show noninvasive viral imaging in human clinical trials (21). For example, the Edmonston strain of measles virus expressing NIS, MV-NIS, has shown promising results in treating multiple myeloma (22, 23). Another study clinically evaluated MV-NIS in drug-resistant ovarian cancer, which showed NIS expression in three of 13 patients and was associated with prolonged progression-free survival (24). A phase 1 trial using a replication-competent adenovirus, expressing two suicide genes along with NIS, showed the safety and feasibility of noninvasive imaging in human prostate cancer (25). Similar safety of noninvasive imaging by vector-mediated exogenous gene expression has been demonstrated in glioma and liver cancer patients (26, 27). Currently, there are 12 active OV clinical trials targeting breast cancer (clinicaltrials.gov). To our knowledge, this is the first study showing successful tracking of an oncolytic vaccinia virus in humans using SPECT imaging studies. We showed enhancement in SPECT imaging at C1D8 in 78% of patients in this first-in-human clinical trial. Thus far in this dose escalation trial, we have treated with low doses of the OV (1x10^5^, 3x10^5^, or 1x10^6^ PFU). The presence of enhancement on SPECT imaging suggests viral replication in the tumor since these doses are too low to show this level of imaging enhancement without replication. Future studies are needed to confirm pathologic evidence of viral replication to correlate with the imaging findings. One hundred percent of patients with injection at lymph node, intramuscular mass, or dermal/subcutaneous nodules had enhancement on SPECT imaging, suggesting successful viral infection and replication in these types of lesions. Fifty percent of the patients with matted dermal disease had uptake on SPECT imaging. Matted dermal lesions pose several therapeutic challenges. There is a concern for limited uptake in the tumor with intralesional injection of matted dermal disease due to fibrosis in the area. The lack of uptake in two patients with matted dermal disease suggests limited viral uptake and lack of replication in these tumors. Further testing is needed to determine the optimal timing for hNIS-based imaging studies in humans undergoing oncolytic virotherapy. Additionally, further testing is needed at higher viral doses to evaluate if the response seen in hNIS imaging studies is more robust with higher doses of OV. We hypothesize that at higher doses of OV, non-injected tumor lesions will also have enhancement on SPECT imaging, showing viral infection and replication at non-injected cancer sites.

hNIS-based imaging can be performed using SPECT or PET imaging studies. SPECT imaging was chosen over PET for this first-in-human trial since SPECT imaging is more widely available and more cost-effective, which would allow for widespread use in future applications and increased ability for repeated imaging studies for continued monitoring of response. The average cost of a PET scan machine is approximately four times the cost of a SPECT scanner. In addition, studies evaluating economic outcomes in patients undergoing noninvasive imaging tests to evaluate coronary artery disease showed a significant decrease in cost burden with SPECT rather than PET imaging (2-year costs with SPECT $3,965, 95% confidence interval [CI]: $3,520 to $4,411 versus PET $6,647, 95% CI: $5,896 to $7,397, p<0.0001) (28). SPECT imaging is nearly five times more available than PET, both in the United States and globally (29). In addition to decreased cost, SPECT imaging is easier to perform due to the increased half-lives of SPECT radiotracers, when compared to PET. For example, Tc-99m and Indium-111 have half-lives of 6 hours and 2.8 days, respectively, compared to PET imaging radiotracers Fluorine-18 (FDG) and Gallium-68 that have half-lives of 110 minutes and 68 minutes, respectively (30). Limitations to SPECT imaging studies include decreased resolution compared to PET imaging and limitations with absolute quantification of signal, making direct comparisons of signal intensity between patients and between scans difficult. Additionally, lack of absolute quantification limits the ability to standardize SPECT scan results between different radiologists and institutions.

Further evaluation is needed to correlate the response on imaging to clinical response and pathologic immune response. This includes future studies to evaluate whether SPECT imaging studies performed after multiple doses of OV are able to correlate with durability of response or disease progression. The ability to monitor response to oncolytic virotherapy using noninvasive techniques earlier in the patient’s treatment is vital to phase I clinical trials and future drug development as it has the potential to guide therapy decisions and combination treatments at a time point that is much earlier than serial staging scans or clinical progression. Next steps include evaluating hNIS-based imaging techniques for monitoring viral replication in intravenous (IV) OV therapies. Our institution is currently conducting an ongoing study NCT05346484: A Study of CF33-hNIS (VAXINIA), an Oncolytic Virus, as Monotherapy or in Combination with Pembrolizumab in Adults with Metastatic or Advanced Solid Tumors, which is an open-label, multicenter, dose-escalation phase I study evaluating the safety of CF33-hNIS OV administered via two routes of administration (IT or IV) either as a monotherapy or in combination with pembrolizumab for patients with advanced solid tumors. It will be exciting to see the hNIS imaging results from this trial’s combination therapy IT arm and IV arms. The ability to track viral replication in IV OV therapy in humans will be revolutionary in monitoring the response to these immunotherapy treatments.

There is the potential for combining hNIS-based imaging with other exciting novel imaging techniques, such as intravenous injection of ^89^Zirconium (^89^Zr)-labeled cys-diabodies, which allows for tracking T-cells *in vivo* using ImmunoPET. Kasten et al. showed CD8+ T-cell infiltration in a glioma mouse model following OV therapy using CD8+ T-cell ImmunoPET (30). The safety of ^89^Zr-based imaging has also been shown in humans in early studies (31, 32). The combination of hNIS-based imaging to track OV and ^89^Zr-labeled T-cell imaging techniques would have the potential to allow simultaneous monitoring of both viral replication and T-cell response using noninvasive techniques. Successful hNIS-based tracking of oncolytic virotherapy in this trial provides exciting evidence for the use of noninvasive monitoring of response to immunotherapy treatments in humans. Further optimization of these techniques could allow physicians to guide treatment decisions and optimize outcomes using noninvasive imaging studies.

## Data Availability

The datasets presented in this study can be found in online repositories. The names of the repository/repositories and accession number(s) can be found below: https://www.clinicaltrials.gov/study/NCT05081492.
